# Molecular mechanism of the spider toxin κ-LhTx-I acting on the bacterial voltage-gated sodium channel NaChBac

**DOI:** 10.3389/fphar.2022.924661

**Published:** 2022-08-04

**Authors:** Zhen Xiao, Yaqi Li, Piao Zhao, Xiangyue Wu, Guoqing Luo, Shuijiao Peng, Hongrong Liu, Cheng Tang, Zhonghua Liu

**Affiliations:** ^1^ The National and Local Joint Engineering Laboratory of Animal Peptide Drug Development, College of Life Sciences, Hunan Normal University, Changsha, China; ^2^ Key Laboratory for Matter Microstructure and Function of Hunan Province, Key Laboratory of Low-dimensional Quantum Structures and Quantum Control, School of Physics and Electronics, Hunan Normal University, Changsha, China

**Keywords:** NaChBac, spider toxin, molecular mechanism, voltage sensor trapping, molecular docking, antagonist

## Abstract

The bacterial sodium channel NaChBac is the prokaryotic prototype for the eukaryotic Na_V_ and Ca_V_ channels, which could be used as a relatively simple model to study their structure–function relationships. However, few modulators of NaChBac have been reported thus far, and the pharmacology of NaChBac remains to be investigated. In the present study, we show that the spider toxin κ-LhTx-1, an antagonist of the K_V_4 family potassium channels, potently inhibits NaChBac with an IC_50_ of 491.0 ± 61.7 nM. Kinetics analysis revealed that κ-LhTx-1 inhibits NaChBac by impeding the voltage-sensor activation. Site-directed mutagenesis confirmed that phenylalanine-103 (F103) in the S3–S4 extracellular loop of NaChBac was critical for interacting with κ-LhTx-1. Molecular docking predicts the binding interface between κ-LhTx-1 and NaChBac and highlights a dominant hydrophobic interaction between W27 in κ-LhTx-1 and F103 in NaChBac that stabilizes the interface. In contrast, κ-LhTx-1 showed weak activity on the mammalian Na_V_ channels, with 10 µM toxin slightly inhibiting the peak currents of Na_V_1.2–1.9 subtypes. Taken together, our study shows that κ-LhTx-1 inhibits the bacterial sodium channel, NaChBac, using a voltage-sensor trapping mechanism similar to mammalian Na_V_ site 4 toxins. κ-LhTx-1 could be used as a ligand to study the toxin–channel interactions in the native membrane environments, given that the NaChBac structure was successfully resolved in a nanodisc.

## Introduction

The mammalian voltage-gated sodium (Na_V_) channels are the molecular determinants of action potential generation and propagation in excitable cells (Hodgkin and Huxley, 1990; Hille, 2001). To date, nine Na_V_ channel genes have been identified. Na_V_1.4 and Na_V_1.5 are expressed mainly in the skeletal muscle and myocardial muscle cells, respectively. Na_V_1.1-Na_V_1.3 and Na_V_1.6-Na_V_1.9 are the dominant subtypes in neurons (Goldin, 1999; Catterall et al., 2005; de Lera Ruiz and Kraus, 2015). Their dysfunction causes hyper- or hypo-excitability of the cell types they are expressed in. The altered excitability leads to myotonia (Na_V_1.4), arrhythmia (Na_V_1.5), epilepsy (Na_V_1.1-1.3, Na_V_1.6), and pain (Na_V_1.7-1.9) (Andavan and Lemmens-Gruber, 2011; Huang et al., 2017). Therefore, pharmacological modulators regulating the activity of Na_V_ channels are valuable drug candidates for treating these diseases (Bagal et al., 2015; Wulff et al., 2019). Topologically, the pore-forming Na_V_ alpha subunit contains 24 transmembrane segments, which could be divided into four homologous domains (DI-DIV). The S1–S4 segments from each domain construct the voltage sensor module (VSM), and the S5–S6 segments make the pore module (PM). The four PMs in DI-DIV form the central Na^+^ conducting pathway, with the four VSMs surrounding the pore (Pan et al., 2018; Pan et al., 2019; Shen et al., 2019; Jiang et al., 2020; Pan et al., 2021). Driven by membrane depolarization, the S4 segments, which carry an array of positively charged arginine/lysine residues move outwardly, the local conformation changes were then transmitted into the central pore, resulting in pore opening (de Lera Ruiz and Kraus, 2015). The tremendous progress in cryo-EM structural studies of mammalian Na_V_ channels has enhanced our understanding of their structure–function relationships (Pan et al., 2018; Pan et al., 2019; Shen et al., 2019; Jiang et al., 2020; Pan et al., 2021; Wisedchaisri et al., 2021).

The prokaryotic Na_V_ channels, counterparts of the mammalian ones in prokaryotes, were proposed as evolutionary ancestors of mammalian Na_V_ and Ca_V_ channels (Charalambous and Wallace, 2011). These channels share the key features of mammalian Na_V_ channels, such as voltage-gating and Na^+^ selectivity. They are involved in specialized functions in prokaryotic organisms, such as motility and chemotaxis (Ren et al., 2001; Ito et al., 2004; Koishi et al., 2004). Structurally, it is assembled by four identical subunits, each of which contains six transmembrane segments, which resembles a single domain of the mammalian Na_V_ channel (Scheuer, 2014). Several prokaryotic Na_V_ channels have been identified, including NaChBac from *Bacillus halodurans*, Na_V_PZ from *Paracoccus zeaxanthinifaciens*, Na_V_SP from *Silicibacter pomeroyi*, and Ns_V_Ba from *Bacillus alcalophilus* (Ren et al., 2001; Koishi et al., 2004; DeCaen et al., 2014). In light of the simplified topological structure and efficient heterologous expression of prokaryotic Na_V_ channels, they serve as ideal models for studying the structure–function relationships of mammalian Na_V_ and Ca_V_ channels. For example, investigating the structures of the prokaryotic Na_V_Ab channel in both the resting and activated states uncovered the likely conserved voltage sensing and gating mechanism in mammalian and prokaryotic Na_V_ channels (Wisedchaisri et al., 2019; Catterall et al., 2020). NaChBac is the first prokaryotic Na_V_ channel identified from *Bacillus halodurans* (Ren et al., 2001). Recently, the structure of a chimeric NaChBac channel containing the domain II S3-S4 loop of Na_V_1.7 channel and complexed to the spider toxin HWTx-IV was resolved at high-resolution in a nanodisc. This structure shed light on understanding the toxin–channel interactions in a natural membrane environment (Gao et al., 2020).

Compared to the mammalian Na_V_ channels, relatively few peptide antagonists have been documented for bacterial Na_V_ channels. Most of these toxins act on both the mammalian and bacterial Na_V_ channels. They are either pore-blockers or gating-modifiers based on their mechanisms of action. The mammalian Na_V_ site 1 modulator, μ-Conotoxin PIIIA, was characterized as a pore-blocker of NaChBac and was suggested to directly occlude the conducting pathway with the side chain of Arg14 (Finol-Urdaneta et al., 2019). We previously purified two potent gating-modifier toxins, JZTx-27 and JZTx-14, from the venom of spider *Chilobrachys jingzhao*. These toxins interact with the S3–S4 loop region of NaChBac and inhibit the channel activity by impeding voltage-sensor activation (Tang et al., 2017; Zhang et al., 2018). A recent study testing the activity of several known peptide modulators of mammalian Na_V_ channels showed that the site 4 toxins GsAF-I, GrTx1, and GsAF-II also effectively inhibit NaChBac currents, possibly by affecting the voltage-dependent gating of the channel (Zhu et al., 2020). Interestingly, the site 3-toxin BDS-I has opposite effects on the bacterial and mammalian Na_V_ channels. BDS-I inhibits NaChBac and activates the human Na_V_1.7 (Zhu et al., 2020). The mechanism of action of these gating-modifier toxins, with the exception of JZTx-27 and JZTx-14, has not been defined to the best of our knowledge. Identifying novel peptide modulators of NaChBac and elucidating their mechanism of action will enhance our understanding of the pharmacology of NaChBac. Such studies will also identify and characterize useful ligands for defining toxin–channel interactions in the native membrane environment with established structural biology approaches (Gao et al., 2020).

In the present study, we identified the spider toxin κ-LhTx-1, which was previously reported as an antagonist of the K_V_4 family voltage-gated potassium channel channels (Xiao et al., 2021), as a novel antagonist of the NaChBac channel. Compared with the other known NaChBac peptide inhibitors, κ**-**LhTx-1 selectively inhibited NaChBac but greatly spared the mammalian Na_V_ channels. Kinetics analysis demonstrated that κ**-**LhTx-1 inhibits NaChBac by preventing voltage-sensor activation. Site-directed mutagenesis showed that phenylalanine-F103 (F103) in the S3–S4 extracellular loop of NaChBac was crucial for interacting with κ**-**LhTx-1. Molecular docking revealed that κ**-**LhTx-1 bound to NaChBac mainly by the hydrophobic interaction, with W27 in toxin being registered with F103 in the channel. This study describes κ**-**LhTx-1, a new peptide modulator of NaChBac. This peptide could be used as a tool for characterizing toxin–channel interactions in a native membrane environment using established structural biology methods.

## Materials and methods

### Toxin

Chemical synthesis and refolding of the linear κ-LhTx-1 was conducted as previously described (Xiao et al., 2021). The purity of synthesized toxins was determined to be >98% by analytic HPLC and MALDI-TOF MS analysis.

### Constructs and site-directed mutations

The cDNA of mammalian Na_V_1.2-1.8 channels was kindly gifted from Professor Theodore R. Cummins (Stark Neurosciences Research Institute, Indiana University School of Medicine, Indianapolis, IN, United States) and subcloned in the pCDNA3.1 or pCMV-blank vectors. The cDNA of Na_V_1.9 was obtained by gene synthesis and cloned into the pEGFP-N1 vector as previously reported (Zhou et al., 2017). The NaChBac and Na_V_PZ expression plasmids were kind gifts from Professor David E. Clapham (Janelia Research Campus, Howard Hughes Medical Institute, Ashburn, VA, United States). NaChBac mutants were made by site-directed mutations. Briefly, the NaChBac plasmid was amplified using a pair of oppositely directed primers harboring the designed mutation site with KOD Fx (TOYOBO Co., Ltd., Osaka, Japan), and the PCR products were treated with FastDigest DpnI (ThermoFisher, Waltham, MA, United States) to remove the methylated template. 10 µl digested product was used to transform 100 µl E. coli DH5α competent cells. Transformants were randomly picked for DNA sequencing to obtain the correct mutants.

### Cell culture and transient transfection

HEK293T and ND7/23 cells were cultured in a DMEM medium, and CHO-K1 in a DMEM-F12 medium, supplemented with 10% FBS and 1% PS (all from Gibco; Thermo Fisher Scientific, Inc., Waltham, MA, United States), in standard cell culture conditions (37°C, 5% CO_2_, saturated humidity). The Na_V_ expression plasmid was co-transfected with pEGFP-N1 plasmid into HEK293T (NaChBac and its mutants, Na_V_PZ and Na_V_PZ/P92F, and Na_V_1.2–1.7 channels) or ND7/23 (Na_V_1.8 channel) cells using Lipofectamine 2000 following manufacturer’s instructions (Invitrogen; Thermo Fisher Scientific, Inc., Waltham, MA, United States). Na_V_1.9-GFP plasmid was transfected into ND7/23 using the X-treme GENE HP DNA Transfection Reagent (Roche, Basel, Switzerland). After 4–6 h transfection, the cells were then seeded onto poly-lysine-coated coverslips. Note that the Na_V_1.9 transfected ND7/23 cells were maintained at 28°C for additional 20 h to promote the functional expression of the channel after the initial culture at 37°C for 24 h.

### Electrophysiology

Whole-cell patch clamp recordings were performed using a MultiClamp 700B amplifier equipped with the Axon Digidata 1550 AD/DA digitizer (Molecular devices, San Jose, CA, United States) at room temperature. The EGFP fluorescence was used to identify the positively transfected cells. The bath solution for sodium currents recording contains (in mM): 140 NaCl, 5 KCl, 1 MgCl_2_, 2 CaCl_2_, 10 Glucose, and 10 HEPES; adjust pH to 7.3 with NaOH. The corresponding pipette solution contains (in mM): 140 CsF, 1 EGTA, 10 NaCl, and 10 HEPES; adjust pH to 7.2 with CsOH. All the chemicals were obtained from Sigma-Aldrich (Sigma-Aldrich, Saint Louis, MO, United States). For Na_V_1.8 and Na_V_1.9 currents recording, 1 μM TTX was added to the bath solution to eliminate the contamination of endogenous TTX-sensitive Na_V_ currents in ND7/23 cells. The recording pipettes were made from glass capillaries using a PC-10 puller (NARISHIGE, Tokyo, Japan), and the pipette resistance was adjusted to be between 2 and 3 MΩ after filling with the pipette solution. Whole-cell configuration was established following a standard program. The fast and slow capacitance was sequentially canceled using a computer-controlled circuit of the amplifier. Moreover, series resistance was compensated by 80% to reduce the voltage error, and the cells with series resistance larger than 10 MΩ after break-in were discarded. The steady-state activation curve of NaChBac channels was obtained by calculating the conductance (G) at each depolarizing voltage (V) using the equation: G = I/(V–V_rev_), and plotting G/G_max_ as a function of V, where I, G_max_, and V_rev_ represent the current amplitude, the maximum conductance, and the reversal potential of the channel, respectively.The G–V curve was fitted using the Boltzmann equation: y = 1/{1+exp[(V_a_–V)/K_a_]}, in which V_a_, V, and K_a_ represent the half maximum activation voltage, test voltage and slope factor, respectively. The steady-state inactivation curve of NaChBac channels was measured using a classical two-pulse protocol: cell was held at −120 mV, and a series of 1 s pre-pulses (−120 to 0 mV, in 10 mV increment) were applied to induce the channel inactivation, followed by a −20 mV/500 ms test pulse to assess the availability of non-inactivated channels (sweep interval was set to 10 s). Currents at the test pulse (I) were normalized to the maximum one (I_max_) and plotted as a function of the conditional voltage (V), the curve was fitted by the Boltzmann equation: I/I_max_ = A + (1−A)/{1 + exp[(V−V_h_)/K_h_]}, where V_h_ is the half-maximum inactivation voltage, A represents the minimum channel availability, and K_h_ is the slope factor. The binding and unbinding kinetics of κ-LhTx-1 with NaChBac were measured by monitoring the time-dependent current rundown after toxin application and recovery upon bath solution perfusion. The association time constant (τ_on_) was calculated by fitting the decay phase with the one exponential decay equation: y = y_steady_+ae^−x/τ^; and the dissociation time constant (τ_off_) was calculated by fitting the recovery phase with one exponential rising equation: y = y_(0)_+a(1−e^−x/τ^). The concentration-response curves were fitted using the following Hill logistic equation: y = f_max_−(f_max_−f_min_)/(1+([Tx]/IC_50_)^n^), where f_max_ and f_min_ represent the maximum and minimum response of the channel to toxin, [Tx] represents the toxin concentration, IC_50_ represents the half maximum inhibition concentration, and n is an empirical Hill coefficient, respectively. Data were acquired using Clampex 10.5 (Molecular devices, San Jose, CA, United States).

### Molecular docking

The structure of κ-LhTx-1 was predicted by C-I-TASSER by using a contact-guided iterative threading assembly refinement (Zheng et al., 2021). The resting-state conformation model of NaChBac channel was generated by modifying the partially activated conformation NaChBac structure (PDB ID: 6VWX) according to the resting-state Na_V_Ab/Na_V_1.7-_V_S2A chimeric channel structure (PDB ID: 7K48) (Wisedchaisri et al., 2021). The binding mode between NaChBac and κ-LhTx-1 was simulated by *ZDOCK* (ZDOCK 3.0.2) (Mintseris et al., 2007; Pierce et al., 2014). The residues which buried into the membrane were blocked in protein-peptide docking. F103 was selected as the binding site residue according to the results of the patch clamp assay.

### Data analysis

Data were presented as the MEAN ± SEM, where *n* represents the number of separate experimental cells. Data were analyzed by using the software Clampfit 10.5 (Axon Instruments, Irvine, CA, United States), Graphpad Prism 5.01 (GraphPad Software, La Jolla, CA, United States), and Excel 2010 (Microsoft Corporation, Redmond, WA, United States). Statistical difference was assessed using the unpaired *t*-test, and the significant difference was accepted at *p* < 0.05.

## Results

### κ-LhTx-1 is a novel NaChBac antagonist

**FIGURE 1:**
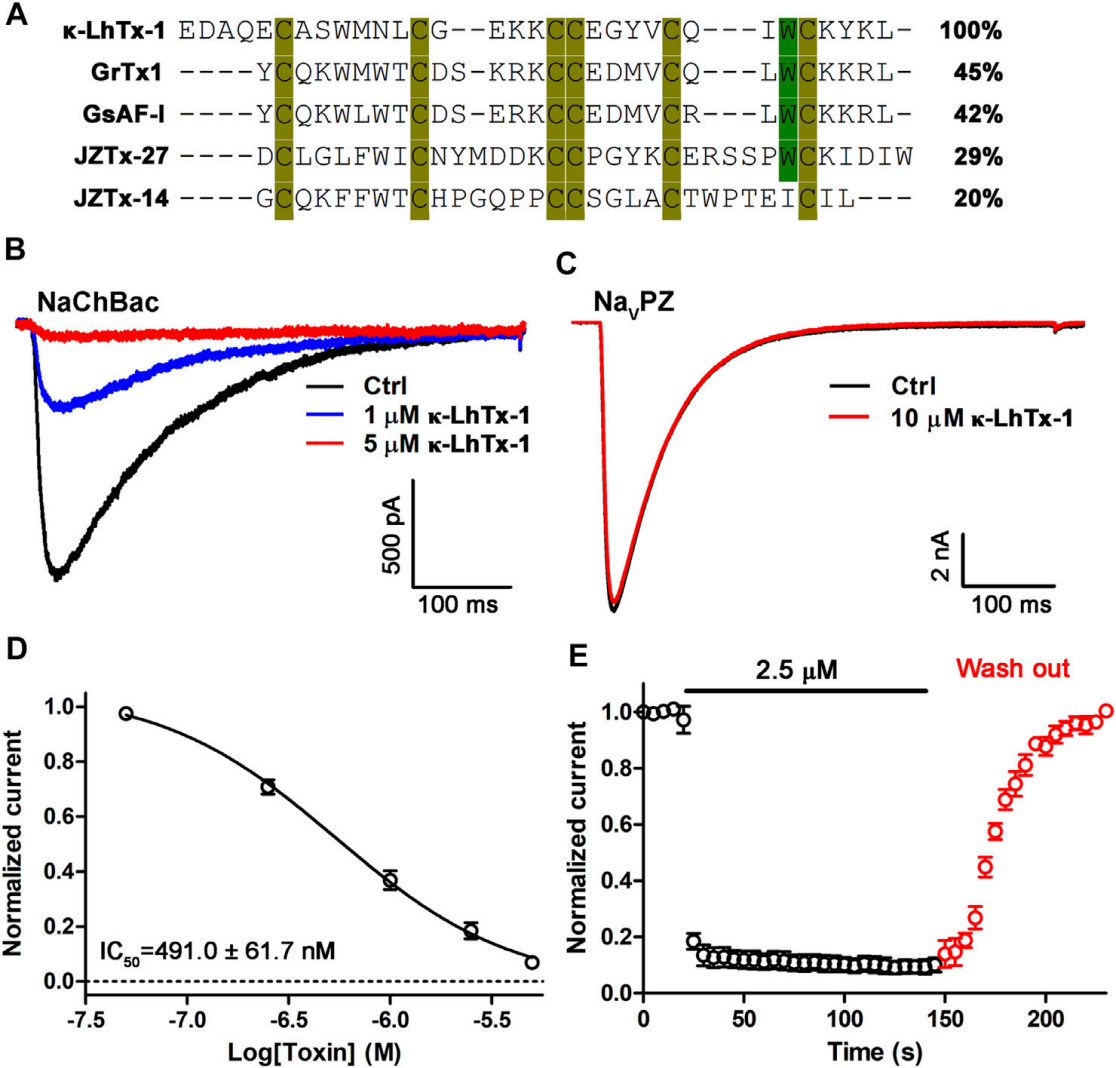
Characterization of κ-LhTx-1 as a novel NaChBac antagonist. **(A)** Sequence alignment of κ-LhTx-1 with other known NaChBac peptide antagonists. **(B)** Representative traces showing κ-LhTx -1 dose-dependently inhibited NaChBac currents elicited by depolarization to −20 mV from the holding potential of −100 mV (*n* = 5). **(C)** Representative traces showing that Na_V_PZ currents were not affected by 10 μM κ-LhTx-1 treatment (*n* = 4). **(D)** Dose-response curve of κ-LhTx-1 inhibiting NaChBac at −20 mV, and the IC_50_ value was determined as 491.0 ± 61.7 nM (*n* = 5). **(E)** Time course of NaChBac current inhibition by 2.5 μM κ-LhTx-1 and subsequent washing off with bath solution. The association time constant (τ_on_) and the dissociation time constant (τ_off_) were determined as 2.0 ± 0.2 s and 34.1 ± 2.5 s, respectively (*n* = 5).

We screened RP-HPLC fractions of the venom of spider *Pandercetes sp* (the lichen huntsman spider) for potential modulators of NaChBac. We identified κ-LhTx-1, an antagonist of K_V_4 family potassium channels, as a potent inhibitor of NaChBac (Figure 1B) (Xiao et al., 2021). The amino acid sequence of κ-LhTx-1 shows relatively low homology to the other known NaChBac peptide inhibitors, including GrTx1 (45%), GsAF-I (42%), JZTx-27 (29%), and JZTx-14 (20%). However, they all share a conserved cysteine framework, which is predicted to fold as an ICK motif (Figure 1A) (Pallaghy et al., 1994). Due to the low abundance of κ-LhTx-1 in the venom, we used the synthetic toxin, which was shown to be correctly refolded as the native one (Xiao et al., 2021), for further experiments. κ-LhTx-1 dose-dependently inhibited the peak current of NaChBac with an IC_50_ of 491.0 ± 61.7 nM at −20 mV (Figures 1B,D). Interestingly, κ-LhTx-1 did not modulate another bacterial sodium channel, Na_V_PZ, even at 10 μM (Figure 1C). Toxin-binding kinetics assays showed that κ-LhTx-1 rapidly inhibited NaChBac currents, with a time constant of 2.0 ± 0.2 s for toxin association. Dissociation of κ-LhTx-1 from NaChBac was slow with a time constant of 34.1 ± 2.5 s (Figure 1E).

### Effects of κ-LhTx-1 on the gating kinetics of NaChBac

**FIGURE 2:**
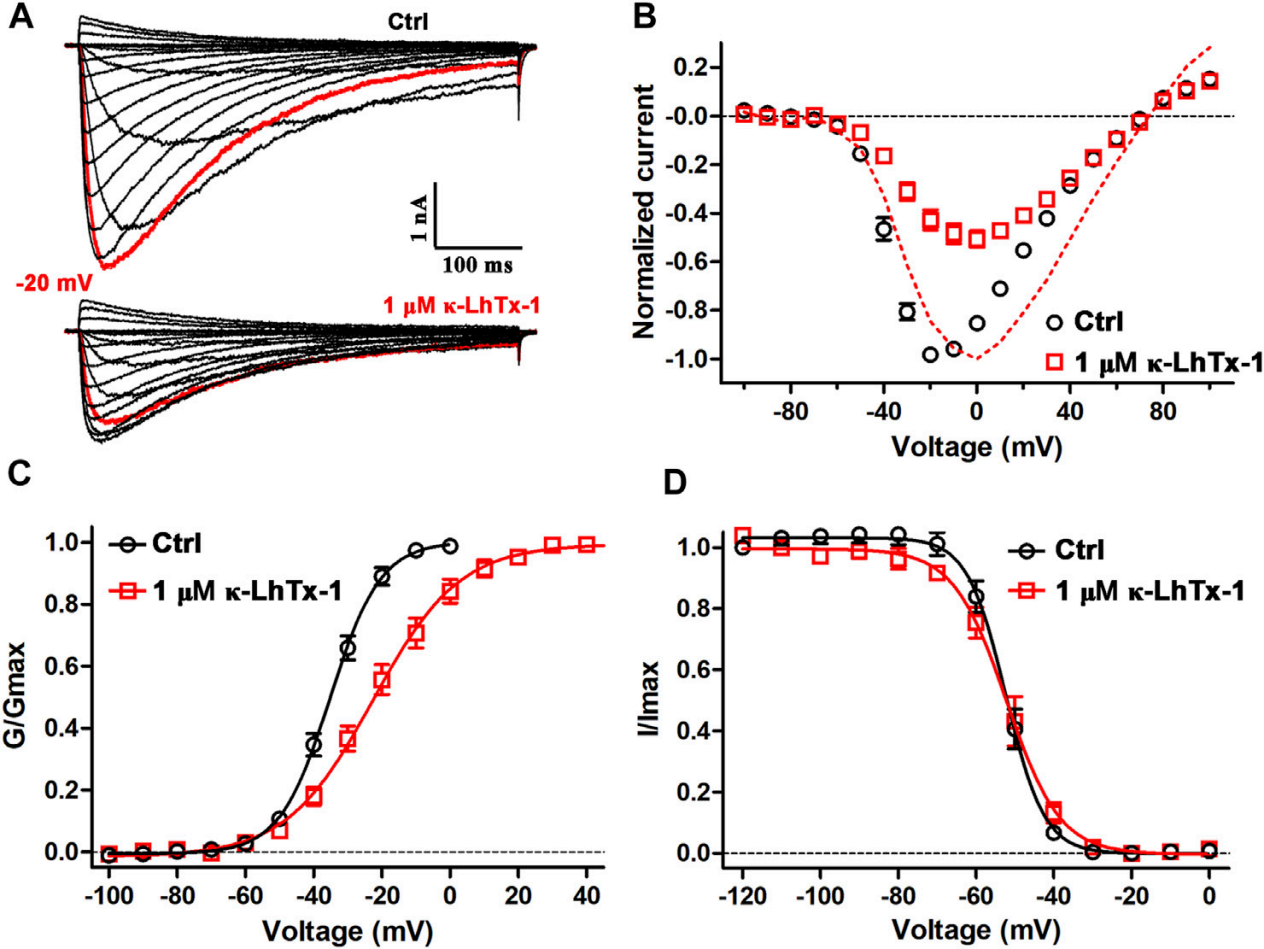
Effects of κ-LhTx-1 on the gating kinetics of NaChBac. **(A)** Representative NaChBac currents before (upper panel) and after (lower panel) 1 μM κ-LhTx-1 treatment. Currents were elicited by step depolarizations from −100 to +100 mV from a holding potential of −100 mV. The red traces show the currents at −20 mV (*n* = 6). **(B)** I–V relationships of NaChBac before and after 1 μM κ-LhTx-1 treatment. The red dashed line shows the I–V relationship of toxin-treated channels by normalizing the currents to their own maximum (*n* = 6). **(C)** Steady-state activation relationships of NaChBac before and after 1 μM κ-LhTx-1 treatment (V_a_ = −35.0 ± 1.3 mV and −22.0 ± 2.8 mV, K_a_ = 7.3 ± 0.4 mV and 12.5 ± 0.9 mV, for control and toxin-treated channels, respectively; *p* < 0.001 when comparing both the V_a_ and K_a_ values between the control and toxin groups; unpaired *t*-test; *n* = 6). **(D)** Steady-state inactivation relationships of NaChBac before and after 1 μM κ-LhTx-1 treatment (V_h_ = −52.3 ± 1.3 mV and −53.0 ± 1.9 mV, K_h_ = −4.6 ± 0.3 mV and −6.0 ± 0.6 mV, for control- and toxin treated channels, respectively; *n* = 5).

The peptide toxins inhibit Na_V_ channels possibly by physically occluding the ion conducting pathway or by impeding the voltage-dependent activation of the voltage sensor, which could be assessed by comparing the channels’ gating kinetics before and after toxin treatment. We first tested the effect of κ-LhTx-1 on the current–voltage (I-V) relationships of NaChBac. Figure 2A shows the representative current traces of the same cell before and after 1 μM κ-LhTx-1 treatment, demonstrating that the inward currents were partially inhibited, while the outward currents were unaffected. This result was further validated by the I–V curves shown in Figure 2B. Between the depolarization voltages of −50 mV and +30 mV, NaChBac currents were differently inhibited by 1 μM κ-LhTx-1, whereas the inhibition was absent at depolarizing voltages higher than +40 mV. These data suggested that those toxin-bound NaChBac channels were reopened as toxin-free channels at stronger depolarizations, resulting in the phenotype of voltage-dependent inhibition. When normalizing the currents at each depolarizing voltage in the toxin-treated group to its maximum peak current, the I–V relationship showed apparent right-forward shift when compared with the control group. Likewise, 1 μM κ-LhTx-1 caused a significant shift of the G-V curve toward more depolarized direction, and the half maximum activation voltage (V_a_) was determined as −35.0 ± 1.3 mV and −22.0 ± 2.8 mV for control and toxin-treated channels, respectively (Figure 2C; *p* < 0.001, unpaired *t*-test). Meanwhile, κ-LhTx-1 treatment also remarkably reduced the slope of the G–V curve (Figure 2C; K_a_ = 7.3 ± 0.4 mV and 12.5 ± 0.9 mV for control and toxin-treated channels, respectively; *p* < 0.001, unpaired *t*-test), indicating toxin greatly attenuated the channel voltage sensitivity during the voltage-dependent activation. These data strongly suggested that κ-LhTx-1 trapped the voltage sensor of the NaChBac channel in a deactivated state, while the energy barrier of voltage sensor outward movement caused by toxin binding could be counteracted by strengthening membrane depolarization. The steady-state inactivation of NaChBac channels, however, was not affected by κ-LhTx-1 (Figure 2D). Altogether, these results argued that κ-LhTx-1 trapped the voltage sensor of the NaChBac channel in the deactivated state, functioning as a gating modifier.

### The molecular determinants in NaChBac for interacting with κ-LhTx-1

**FIGURE 3:**
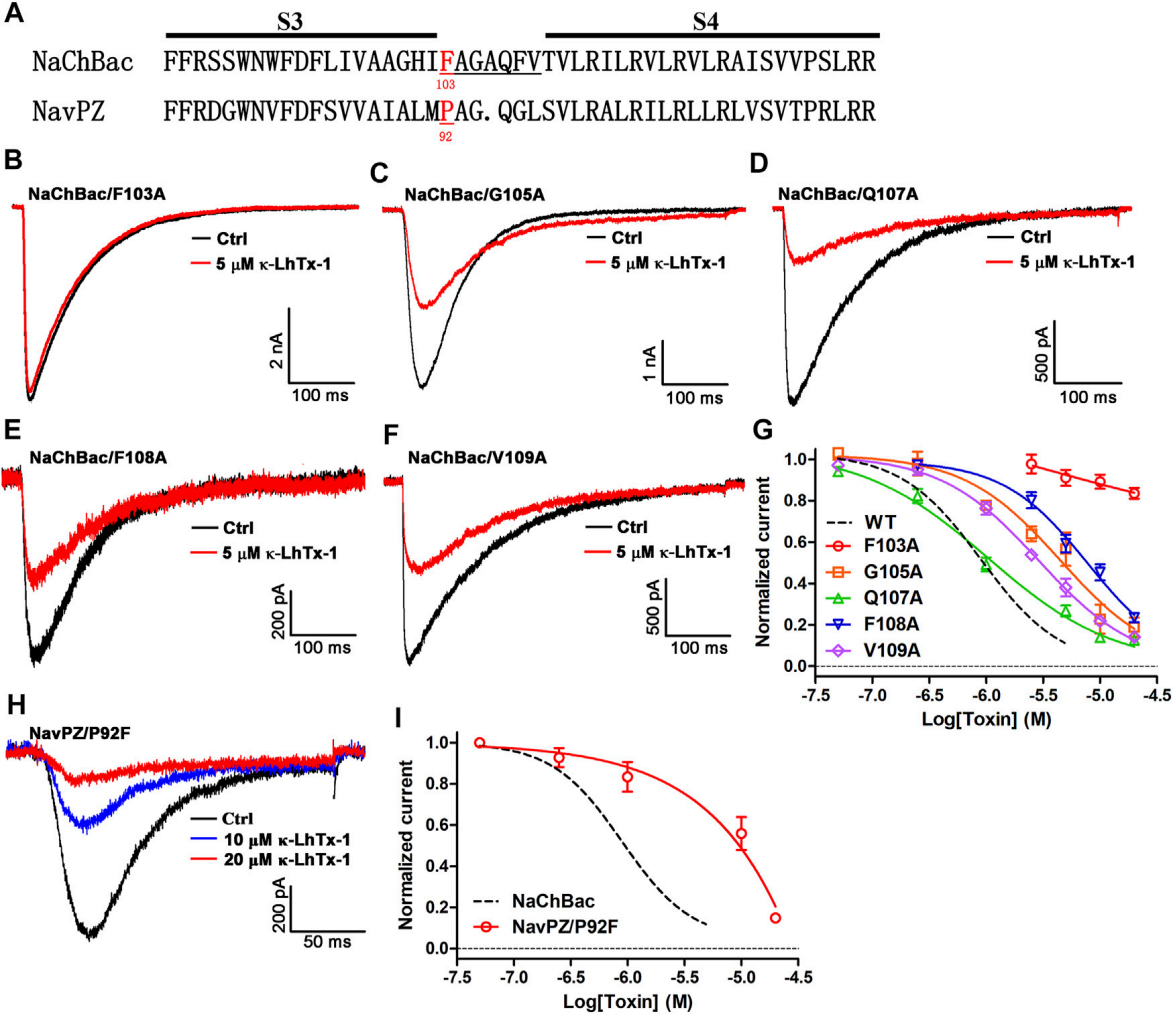
Key molecular determinants in NaChBac for interacting with κ-LhTx-1. **(A)** Sequence alignment of the NaChBac and Na_V_PZ, the number below the residue indicates its location in the sequence. **(B)** Representative traces showing F103A mutation in NaChBac almost fully abolished κ-LhTx-1 inhibition on the channel (*n* = 5). **(C**–**F)** Representative traces showing the inhibitory effects of 5 μM κ-LhTx-1 on NaChBac mutants as illustrated (*n* = 5–6). **(G)** Dose-response curves of κ-LhTx-1 inhibiting the NaChBac mutant channels, and the IC_50_ values were determined as 5.5 ± 1.4 μM, 1.0 ± 0.1 μM, 7.8 ± 1.0 μM, and 2.6 ± 0.4 μM for NaChBac/G105A, NaChBac/Q107A, NaChBac/F108A, and NaChBac/V109A, respectively (*n* = 5–6). The black dashed line shows the dose-response curve of κ-LhTx-1 against the wild-type NaChBac channel. **(H)** Representative traces showing the κ-LhTx-1 dose-dependently inhibited Na_V_PZ/P92F currents elicited by depolarization to +30 mV from the holding potential of −100 mV (*n* = 5). **(I)** Dose-response curve of κ-LhTx-1 inhibiting the currents of Na_V_PZ/P92F at +30 mV, the IC_50_ values were determined as 6.7 ± 2.3 μM (*n* = 4).

Since κ-LhTx-1 is a gating-modifier of NaChBac, we wondered if it might bind to the channel’s S3–S4 extracellular loop. The protein sequence-alignment through the S3–S4 region of the κ-LhTx-1-sensitive NaChBac channel and the κ-LhTx-1-resistant Na_V_PZ channel is shown in Figure 3A. The alignment in Figure 3A highlights F103 in NaChBac and the corresponding Proline-92 (P92) in Na_V_PZ. We used an alanine scan strategy to identify the key residues for interacting with κ-LhTx-1 in this region (Figure 3A). All these NaChBac mutants were functionally expressed in CHO-K1 cells (Figures 3B–F). We tested the inhibitory effect of κ-LhTx-1 on each mutant channel at voltage eliciting its maximum inward peak current. The data showed that F103A, G105A, F108A, and V109A mutations in NaChBac all attenuated the inhibitory effect of κ-LhTx-1, with 5 µM toxin not affecting the currents of NaChBac/F103A and only partially inhibiting the currents of NaChBac/G105A, NaChBac/F108A, and NaChBac/V109A by approximately 43.4 ± 8.1%, 40.7 ± 4.2%, and 64.4 ± 4.1%, respectively (Figures 3B,C,E,F). The activity of κ-LhTx-1 on NaChBac/Q107A was not changed a lot when compared with that of the wild type channel (Figure 3D). The dose–response curves in Figure 3G showed that κ-LhTx-1 inhibited the currents of NaChBac/G105A, NaChBac/Q107A, NaChBac/F108A, and NaChBac/V109A with an IC_50_ of 5.5 ± 1.4 μM, 1.0 ± 0.1 μM, 7.8 ± 1.0 μM, and 2.6 ± 0.4 μM, respectively, (Figure 3G). The IC_50_ for NaChBac/F103A could not be determined from the curve as 20 μM κ-LhTx-1 only inhibited its currents by 16.4 ± 2.6% (Figure 3G). Next, we tried to convert the κ-LhTx-1-resistant Na_V_PZ channel into the κ-LhTx-1-sensitive channel. Since F103 in NaChBac was deemed to be important for κ-LhTx-1 modulation, we decided to replace the corresponding residue in Na_V_PZ (P92) with phenylalanine. The Na_V_PZ/P92F mutant was potently inhibited by κ-LhTx-1 (Figure 3H), with an IC_50_ of 6.7 ± 2.3 μM (Figure 3I). Our successful conversion of κ-LhTx-1-resistant Na_V_PZ into a sensitive channel strongly supports the importance of F103 in NaChBac and the corresponding P92 in Na_V_PZ.

### Effects of κ-LhTx-1 on mammalian Na_V_ channels

**FIGURE 4:**
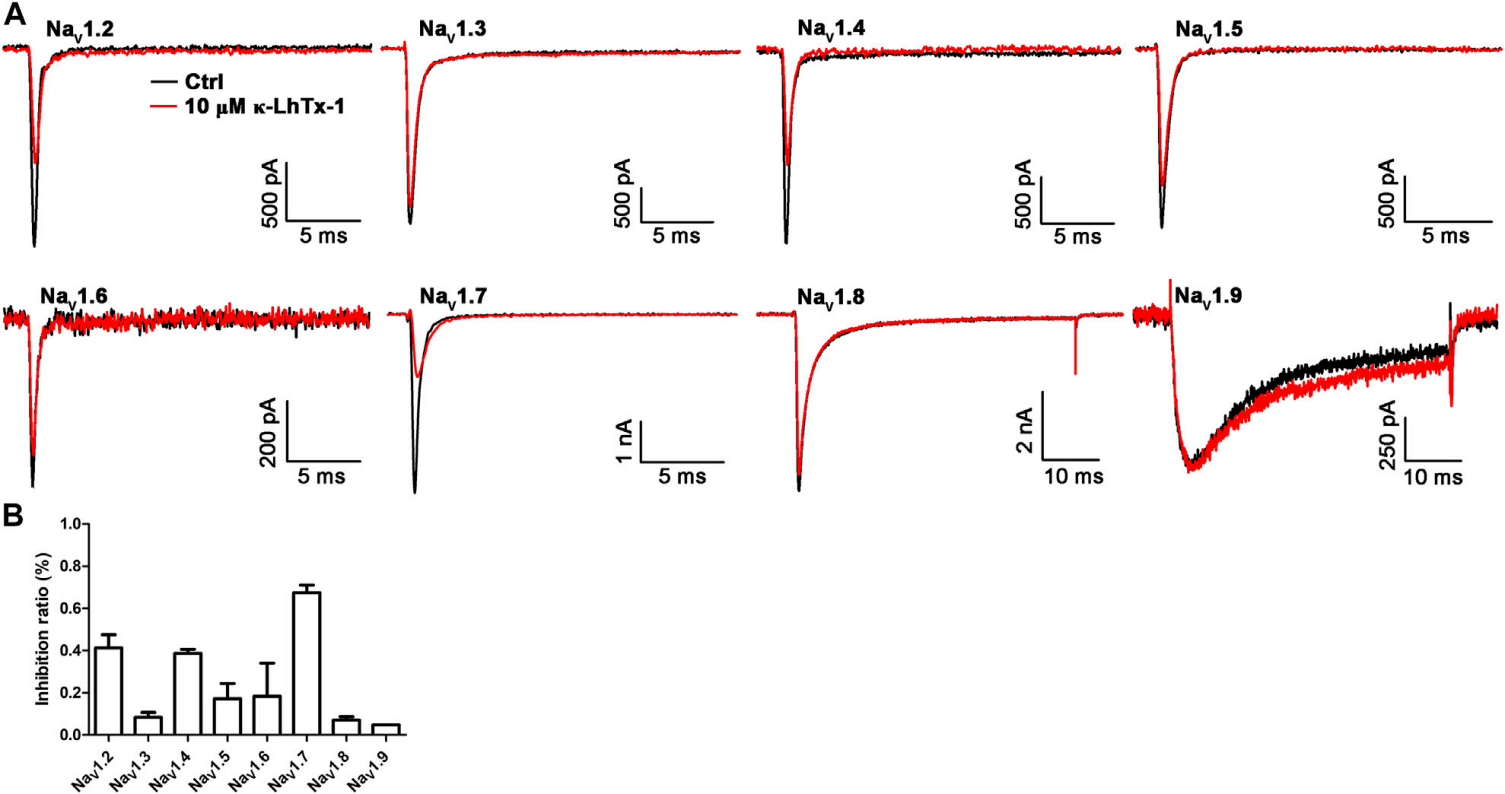
Effects of κ-LhTx-1 on the mammalian Na_V_ channels. **(A)** Representative traces showing the effect of 10 μM κ-LhTx-1 on Na_V_1.2–1.9 channels (*n* = 3–5). **(B)** Bar graph showing the inhibition of 10 μM κ-LhTx-1 on the peak currents of Nav1.2–1.9 channels. The inhibition ratio was determined as 41.3 ± 6.2%, 8.4 ± 2.3%, 38.7 ± 1.9%, 17.2 ± 7.3%, 18.4 ± 15.7%, 67.4 ± 3.7%, 7.1 ± 1.5%, and 4.8 ± 0.1% for Na_V_1.2, Na_V_1.3, Na_V_1.4, Na_V_1.5, Na_V_1.6, Na_V_1.7, Na_V_1.8, and Na_V_1.9 channels, respectively (*n* = 3–5).

The biological activity of κ-LhTx-1 on the mammalian K_V_ channels has been systematically examined, which showed it potently inhibited the K_V_4 family potassium channels without affecting other K_V_ channels, including K_V_1.1, K_V_1.3-1.5, K_V_2.1, and K_V_3.1-3.4 (Xiao et al., 2021). Herein, we tested the activity of κ-LhTx-1 on the mammalian Na_V_ channels. As shown in Figures 4A,B, 10 μM κ-LhTx-1 showed weak inhibition on Na_V_1.3 (8.4 ± 2.3%), Na_V_1.5 (17.2 ± 7.3%), Na_V_1.6 (18.4 ± 15.7%), Na_V_1.8 (7.1 ± 1.5%), and Na_V_1.9 (4.8 ± 0.1%) channels and relatively stronger inhibition on Na_V_1.2 (41.3 ± 6.2%), Na_V_1.4 (38.7 ± 1.9%), and Na_V_1.7 (67.4 ± 3.7%) channels. Compared with the potent inhibition of κ-LhTx-1 on the K_V_4 family potassium channels and NaChBac channels, the toxin showed much weaker activity on the mammalian Na_V_ channels.

### The toxin–channel interface as revealed by molecular docking

**FIGURE 5:**
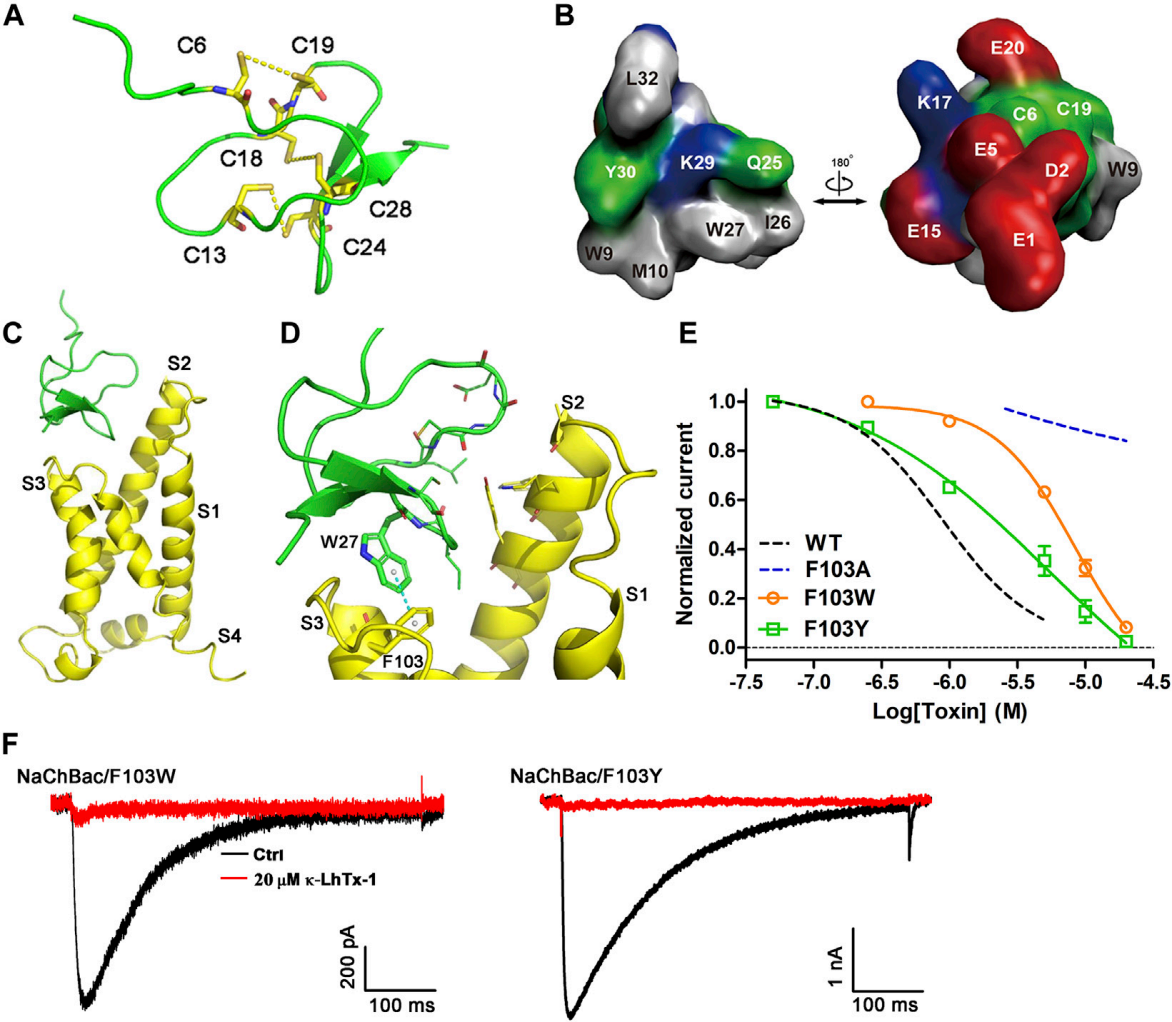
Molecular docking of κ-LhTx-1 with NaChBac. **(A)** Predicted structure of the κ-LhTx-1 by *C-I-TASSER,* the peptide toxin GsMTx2 (PDB ID: 1LUP) was used as the template. Cysteines were highlighted in yellow, and the predicted disulfide bonds were shown (C_6_ - C_19_, C_13_ - C_24_, and C_18_ - C_28_). **(B)** Surface presentation of κ-LhTx-1 structure. Positively charged residues are shown in blue, negatively charged residues in red, hydrophobic residues in gray, and polar uncharged residues in green. **(C)** Docked κ-LhTx-1-NaChBac complex with the highest score. The resting-state NaChBac channel and the κ-LhTx-1 toxin were shown in yellow and green, respectively. For simplicity, only the S1–S4 region of a single channel subunit was shown. **(D)** Enlarged view of the interaction interface between NaChBac and κ-LhTx-1, in which W27 in toxin captured the experimentally determined key residue in NaChBac, F103, in the toxin–channel interface. **(E)** Dose-response curve of κ-LhTx-1 inhibiting the NaChBac mutants as shown, and the IC_50_ values were determined as 9.5 ± 2.9 μM and 3.7 ± 1.5 μM for NaChBac/F103W and NaChBac/F103Y, respectively. The black and red dashed lines show the dose-response curves of κ-LhTx-1 against the wild-type NaChBac and NaChBac/F103A mutant channel, respectively (*n* = 5). **(F)** Representative traces showing the inhibition effect of 20 μM κ-LhTx-1 on the currents of NaChBac/F103W and NaChBac/F103Y mutant channels (*n* = 5).

The κ-LhTx-1 structure was simulated by C-I-TASSER (Figure 5A). The six cysteines in toxin formed three disulfide bonds as C6-C19, C13-C24, and C18-C28 (number indicates the position of cysteines in toxin sequence). Analyzing the solvent-accessible surface of κ-LhTx-1 showed it is an amphilic molecule, in which most hydrophobic residues are mainly distributed on one side, forming a hydrophobic patch, whereas charged residues are distributed on the other side, forming a hydrophilic surface (Figure 5B). The toxin–channel docking complex is shown in Figure 5C. The modeled resting-state of NaChBac and κ-LhTx-1 is depicted in yellow and green, respectively. The best scoring docking complex showed that the toxin inserted as a wedge into the cleft between S1–S2 and S3–S4 loops (Figure 5C), resembling that of α-scorpion toxin interacting with the voltage sensor of the mammalian Na_V_ channel (Wang et al., 2011; Clairfeuille et al., 2019). In this docking model, F103 in NaChBac and W27 in κ-LhTx-1 were captured in the channel–toxin interface, in which W27 was registered with F103 by the π–π stacking interaction (Figure 5D). We used site-specific mutagenesis to experimentally verify the functional importance of the phenyl ring of F103 for κ-LhTx-1 binding. The F103A mutation abolished the modulation of NaChBac by κ-LhTx-1 (Figures 3B, 5E). Replacement of F103 with aromatic residues (F103W, F103Y) retained the inhibition by κ-LhTx-1, albeit with lower potency than the wild-type channel. These mutants were inhibited by κ-LhTx-1 with IC_50s_ of 9.5 ± 2.9 μM for NaChBac/F103W (∼19-fold lower) and 3.7 ± 1.5 μM for NaChBac/F103Y (∼8-fold lower) (Figures 5E,F). Taken together, these results argued that the phenyl ring of F103 in NaChBac played a critical role in binding with κ-LhTx-I, and the association between κ-LhTx-1 and NaChBac likely relies on a hydrophobic interaction.

## Discussion

κ-LhTx-1 was demonstrated in our previous study to selectively inhibit the mammalian K_V_4 family potassium channels (Xiao et al., 2021). The present study confirmed that κ-LhTx-1 is also a novel NaChBac antagonist. The G–V curve of NaChBac was remarkably shifted to depolarized direction by κ-LhTx-1, indicating κ-LhTx-1 acts on NaChBac as a gating-modifier trapping the deactivated voltage sensor. Moreover, the site-directed mutagenesis and molecular docking showed κ-LhTx-1 bound to the S3–S4 loop of NaChBac, with the phenylalanine-103 (F103) being the most critical residue. Molecular docking also confirmed the W27 residue in κ-LhTx-1 was registered with F103 in NaChBac, likely by the π–π stacking interaction. The action mode of κ-LhTx-1 on NaChBac and its binding site on the channel resembles that of JZTx-27 (Tang et al., 2017). However, unlike JZTx-27, κ-LhTx-1 slightly inhibited the activation but not the inactivation of mammalian Na_V_ channels. The structure of chimeric NaChBac channel harboring the DII S3-S4 linker of Na_V_1.7 channel in complex with the spider toxin HwTx-IV in a nanodisc has been successfully resolved, which has showed greatly improved resolution of the toxin–channel interacting interface and enabled visualization of their binding details (Gao et al., 2020). κ-LhTx-1 in the present study thus provided another useful ligand for studying the toxin and wild-type NaChBac interaction in the native membrane environments.

Historically, NaChBac was shown to pharmacologically resemble the mammalian Na_V_ and Ca_V_ channels, as revealed by its inhibition by lots of Ca_V_ and Na_V_ channel modulators, such as lidocaine, nifedipine, and various peptide toxins (Ren et al., 2001; Lee et al., 2012; Tang et al., 2017; Zhang et al., 2018; Zhu et al., 2020). κ-LhTx-1 was previously shown to inhibit the K_V_4 family potassium channels in a voltage-dependent manner (Xiao et al., 2021). Consequently, the present study provided the first example that NaChBac also shares similar pharmacology properties with the K_V_ channels. Gating currents analysis confirmed that κ-LhTx-1 trapped the voltage sensor of K_V_4 channels in a deactivated state (Xiao et al., 2021). Therefore, we concluded that κ-LhTx-1 inhibited the K_V_4 and NaChBac channels using the same voltage-sensor trapping mechanism.

Mechanistically, the association between gating-modifier toxins and ion channels relies on electrostatic force and/or hydrophobic interaction between their interface (Bosmans et al., 2008). As that of the HpTx2 binding with the K_V_4.3 channel solely by hydrophobic force (Zhang et al., 2007; DeSimone et al., 2009), κ-LhTx-1 might also interact with NaChBac mainly by a hydrophobic interaction, as revealed by mutation and molecular docking analyses. Indeed, hydrophobic residues on the S3–S4 loop of NaChBac have been commonly characterized as key molecular determinants in interacting with its gating-modifier toxins, such as F103 for κ-LhTx-1 and JZTx-27 and F108 for JZTx-14 (Tang et al., 2017; Zhang et al., 2018). Interestingly, the hydrophobic residues LF/LV in the S3b region of K_V_4 channels were also identified as the key binding determinants for κ-LhTx-1 (Xiao et al., 2021), and it can be rationally presumed that hydrophobic interactions also underlie the interaction between κ-LhTx-1 and K_V_4 channels. Additionally, sequence alignment revealed that κ-LhTx-1 and other NaChBac peptide antagonists from spider venom share a conserved cysteine framework, and the critical W27 residue in κ-LhTx-1 is also conserved in most of them (Figure 1A, except for JZTx-14); therefore it is reasonable to speculate that these toxins used this conserved W residue to interact with NaChBac *via* hydrophobic interaction. Indeed, molecular docking analysis in our previous study also showed that this W residue in JZTx-27 interacts with F98 in another bacteria sodium channel, Ns_V_Ba (Tang et al., 2017).

## Data Availability

The original contributions presented in the study are included in the article/Supplementary Material; further inquiries can be directed to the corresponding authors.
